# Measurement of Rectal Diameter and Anterior Wall Thickness by Ultrasonography in Children with Chronic Constipation

**DOI:** 10.5152/tjg.2022.22165

**Published:** 2022-12-01

**Authors:** Güzide Doğan, Merter Keçeli, Sibel Yavuz, Adem Topçu, Erhun Kasırga

**Affiliations:** 1 Department of Pediatric Gastroenterology, Hepatology and Nutrition, Bezmialem Vakıf University Faculty of Medicine, İstanbul, Turkey; 2 Department of Radiology, Celal Bayar University Faculty of Medicine, Manisa, Turkey; 3 Department of Pediatric Gastroenterology, Adıyaman University Training and Research Hospital, Adıyaman, Turkey; 4 Department of Radiology, ealth Sciences University Haseki Training and Education Hospital, İstanbul, Turkey; 5 Department of Pediatric Gastroenterology, Hepatology and Nutrition, Celal Bayar University Faculty of Medicine, Manisa, Turkey

**Keywords:** Anterior wall thickness, children, constipation, rectal diameter

## Abstract

**Background:**

Measurement of rectal diameter by ultrasonography helps the clinician in the diagnosis of chronic constipation in children for whom rectal examination cannot be performed. The aim of the study is to determine the rectal diameter and anterior wall thickness values in constipated and healthy children and to evaluate the feasibility of ultrasonography in the diagnosis of functional constipation in children who refuse digital rectal examination.

**Methods:**

One hundred forty constipated and 164 healthy children participated in the study. All patients were divided into 4 subgroups according to their ages (≤3 years [group I], 3.1-6 years [group II], 6.1-12 years [group III], and >12 years [group IV]) and were referred to the radiology department. The measurement was made from above the symphysis pubis, under the ischial spine, and at the bladder neck. Anterior wall thickness measurement was performed. The measurements were recorded according to the presence or absence of fecal mass in the rectum.

**Results:**

Constipated children with fecal mass positive group III was found to have significant difference in all of the planes in rectal diameter measurement. Rectum anterior wall thickness measurement was found to be higher in constipated patients with fecal mass (+) compared to the control. Its measurements in constipated patients in group II, group III, and group IV with no fecal mass were found to be statistically higher than the control group.

**Conclusion:**

The measurement of rectal diameter and anterior wall thickness by ultrasonography as a noninvasive method was performed in children who did not want the digital rectal examination, and it may be useful in the diagnosis of constipation.

**Main Points**

- The measurement made under the ischial spine plane in children aged 6.1-12 years using the US technique may be useful in the diagnosis of chronic constipation.
- Rectal anterior wall thickness measurement using the US technique is a noninvasive method performed in children older than 3 years who do not want to have a digital rectal examination.

## Introduction

Children with constipation frequently visit the pediatrician. The etiology of constipation is mostly functional constipation and a small proportion is due to organic causes.^1-4^ While detailed history and physical examination are routinely recommended in the diagnosis of constipation, abdominal radiography, transabdominal recto-ultrasonographic examination, colonic transit time, rectal biopsies, and colonic manometry are not routinely recommended.^5-8^ Fecal retention is found in 40%-100% of children with functional constipation.^9^ Due to fecal retention, rectum diameter may increase and megarectum may develop.^10^

Digital rectal examination is a part of physical examination in children with chronic constipation. However, the rectal examination cannot always be performed due to reasons such as fear of the examination in young children, an embarrassment in adolescents, and sometimes the families not giving permission. On the other hand, ultrasonographic rectal diameter measurement is a non-invasive method that provides an idea of fecal impaction.^11,12^ Rectal diameter measurement can assist physicians in managing diagnosis and treatment for children and families who do not allow digital rectal examination.

The aim of our study is to determine the rectal diameter and anterior wall thickness values of children with functional constipation, to compare them with the values of healthy children, and to evaluate the feasibility of ultrasonography (US) in the diagnosis of functional constipation in children for whom digital rectal examination cannot be performed.

## Materials and Methods

**Figure 1.**
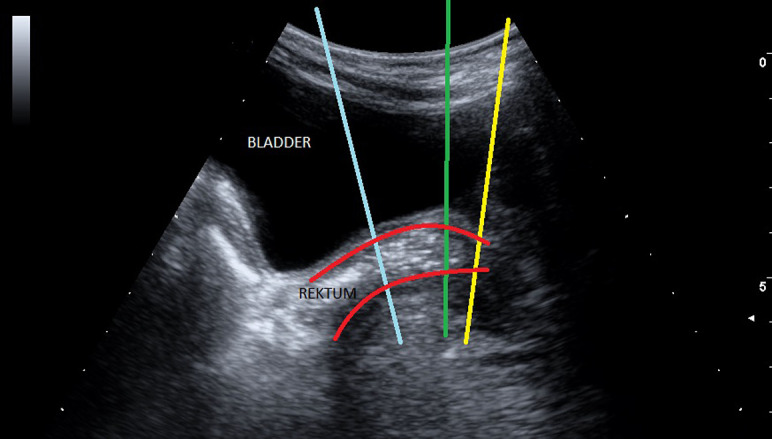
Five-year-old boy. Image obtained from the sagittal plane of the pelvis midline shows the rectum between 2 red lines. The echogenic area seen in the middle section shows the rectum mucosa and fecal material in the lumen. Blue line: Imaging plane passing through the symphysis level. Green line: Imaging plane passing through the ischial spine. Yellow line: Imaging plane passing through the bladder neck level.

**Figure 2.**
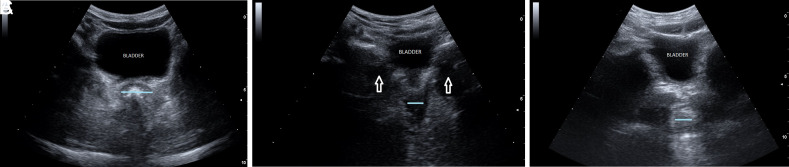
Five-year-old boy. (A) In the axial plane, the US image taken at the symphysis level, the rectum mediolateral diameter (blue line) was measured from outer wall to outer wall. (B) The diameter of the rectum (blue line) was determined by a similar method at the level of the ischial spine. (C) The white arrows show the areas with acoustic shadow created by the spine. The transducer angles downwards. When the ischial spine echo disappears, the bladder neck is reached. The rectum diameter was measured from this level (blue line).

**Figure 3.**
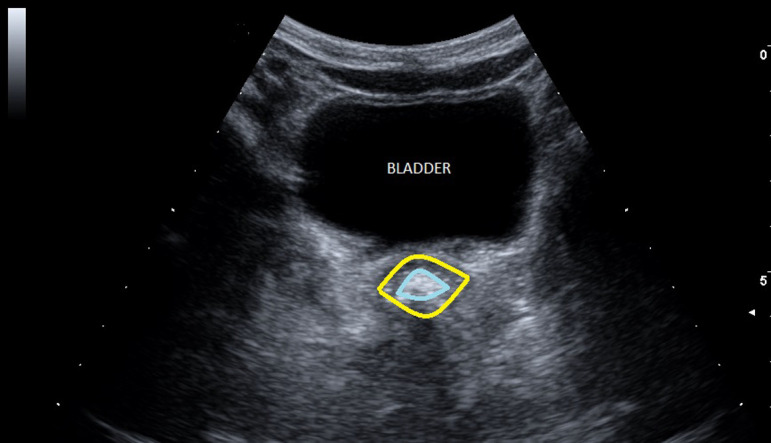
Five-year-old boy. Axial plane USG images at bladder neck. The inner wall (blue line) and outer wall (yellow line) of the rectum are shown. The rectum wall thickness was measured from the front wall near the bladder.

One hundred forty children aged between 6 months and 18 years, who presented to the pediatric gastroenterology division and were diagnosed with functional constipation according to the Rome IV criteria, were included in the study as the patient group. A total of 164 children who did not have chronic constipation and defecated every day or once in 2 days but underwent US examination for a different reason were included in the study as a control group. Children were divided into 4 subgroups according to their ages: ≤3 years (group I), 3.1-6 years (group II), 6.1-12 years (group III), and >12 years (group IV). We used a prospective study design. Children were excluded from the study if they had congenital anomalies of the anorectal region or Hirschsprung disease; if they had disorders such as cerebral palsy, spina bifida, hypothyroidism, diabetes mellitus, or diabetes insipidus; and if they had previously undergone abdominal surgery. A demographic data form containing the information of the patients (age, sex, additional symptoms, defecation frequency, and symptom duration) was filled out. After the examinations in the pediatric gastroenterology outpatient clinic, the participants were sent to the radiology department. Each participant was examined by US without any sedation. The participant was asked to have urine in the bladder to create a viewing window in the pelvis. Considering the possibility of the bladder pressing on the rectum from the front, however, care was taken to prevent bladder distension. The patients were instructed to urinate for maintaining a roughly constant amount of urine in the bladder. The examination was performed by radiologists in a supine position using a 4-MHz curved array transducer (Siemens Acuson ×300, Siemens Health Care, Munich, Germany). Rectum evaluation was started transabdominally with a transducer placed on the anterior abdominal wall of the midline. The measurement was made from the following points by marking from outer wall to outer wall (Figure 1).^1^ Above the symphysis pubis (Figure 2a): The transducer was applied perpendicular to the anterior abdominal wall at the upper edge of the symphysis pubis.^2^ Under the ischial spine (Figure 2b): The transducer on the symphysis was angled toward the pelvis and the ischial spine level was determined by the echogenic appearance of the ischial spine and the detection of the acoustic shadow area behind it.^3^ At the bladder neck (Figure 2c): The transducer was readjusted to a downward angle following the detection of the rectum under the ischial spine, and rectal wall thickness measurement was performed at the level of the bladder neck from the anterior wall (Figure 3). The measurements obtained were recorded. During the examination, the presence or absence of fecal material in the rectum was noted.

This study was conducted in conformity with the Declaration of Helsinki and was approved by the Celal Bayar University Faculty of Medicine Non-Interventional Clinical Research Ethics Committee (November 23, 2016; 20478486-390), and written informed consent was obtained from parents or legal guardians.

### Statistical Analysis

When descriptive statistics (number, percentage distribution, mean, standard deviation, etc.) satisfied normal distribution conditions as evaluated by the Kolmogorov–Smirnov and Shapiro–Wilk tests, the *t*-test was used in independent groups in the comparison of 2 groups in terms of numerical values. When normal distribution was not verified, comparisons were performed with the Mann–Whitney *U* test. The chi-square test was used in the comparison of categorical data. Pearson’s correlation analysis was also performed. Sample size was calculated with power analysis. When α = 0.05 was taken, a minimum of 138 patients were calculated for every group with a power of 85% and effect size (*d*) = 0.30.

## Results

Three hundred four patients were included in the study. Sociodemographic features of the study group are shown in Table 1. The frequency of defecation was 4.62 ± 2.30 days in the constipated group and 1.19 ± 0.39 days in the control group. Encopresis was present in 24 (17.1%) patients, urinary incontinence in 14 (10.0%) patients, and recurrent urinary tract infection in 17 (12.1%) patients. On examination, the anal fissure was detected in 38 (27.1%) patients and skin tags in 19 (13.6%) patients.

Rectal diameter measurements for the patients who were grouped as presence/absence of fecal mass were performed above the symphysis pubis, under the ischial spine, and at the bladder neck and are summarized in Tables 2, 3, and 4, respectively.

At the symphysis pubis plane, the rectal diameter measurement of constipated patients with fecal mass positive was found to be significantly greater in group II and III than the control group (*P* = .04 and *P* = .003, respectively). In group III, fecal mass negative constipated patients’ rectal diameter was found to be greater than in the control group (*P* = .05) (Table 2).

At the ischial spine plane, rectal diameter of constipated children in group III with fecal mass positive or negative was found to be statistically greater than in the control group (*P* = .03 and *P* = .04, respectively) (Table 3).

At the bladder neck plane, the rectal diameter of constipated children with fecal mass positive was found significantly higher in groups II and III than the control group (*P* = .05 and *P* = .001, respectively). There was no statistically significant difference between the fecal mass negative constipated groups and controls (Table 4).

Rectal anterior wall thickness was found to be significantly larger in fecal mass positive constipated patients in group III compared to the control group (*P* = .000). The thickness of the rectum anterior wall of fecal mass negative constipated patients in groups II, III, and IV was found to be significantly larger than that of control group patients (*P* =.02, *P* = .001, and *P* = .000, respectively) (Table 5). It was found that with the prolongation of constipation duration, the thickness of the anterior rectal wall increased (*r* = 0.40, *P *= .000).

## Discussion

Constipation is one of the most common reasons for patients visiting the pediatric gastroenterology department. Digital rectal examination is the part of constipation examination that sometimes has low feasibility. In our study, we found that the measurements at the ischial spine plane in children aged 6.1-12 years with no fecal mass group were statistically significant and the rectum anterior wall thickness was significantly larger in children aged 3.1-6 years and 6.1-12 years than the control groups. Diseases such as encopresis, urinary incontinence, and recurrent urinary tract infections may accompany chronic constipation in children.^2,4^ In this study, the encopresis rate was 17% in the constipated group, and this rate was found to be similar to that of the literature.^6^ In another study conducted in Turkey, the coexistence of encopresis was reported at a rate of 51.7% in chronically constipated children.^13^ The lower incidence of encopresis in our study may be due to the increased awareness of families in Turkey about chronic constipation compared to previous years, with the treatment of children with encopresis having improved. Nephrological problems such as urinary incontinence and recurrent urinary tract infections were also detected in our constipated patients, as the same in the literature.^14^ In a study evaluating the clinical findings of chronically constipated children, the rate of anal fissure as 7.2% and 26.9%.^13,15^ Thus, our data are similar to those of other studies carried out in Turkey (27.1%).

Prolonged fecal retention in constipated children causes megarectum development. Various techniques are used in the radiological evaluation of megarectum and constipation.^13^ The contrast enema technique is difficult to apply in children due to the radiation risk and the invasiveness of the procedure. Fecal impaction may be detected most accurately via digital rectal examination. However, many constipated children and their parents find this procedure unpleasant. Recently, measurement of the rectal diameter via US was reported as a noninvasive diagnostic tool for childhood functional constipation. Di Pace et al^16^ reported that pelvic ultrasound was a quick and child-friendly investigation that could be used to document the presence of megarectum.

Studies have shown that children with chronic constipation have larger rectal diameters than healthy children. In a study conducted in 82 healthy children and 95 children with chronic constipation, rectal crescent size was measured as 2.4 cm in healthy children and 3.4 cm in constipated children, and this was statistically significant. The researchers reported that they used a cut-off point of 3.0 cm for defining megarectum in children.^17^ In a study conducted in Turkey, the rectal diameter of constipated children was evaluated when the bladder was empty and full. It was concluded that it was more meaningful to evaluate the rectum diameter when the bladder was empty, and it was shown that the rectum wall thickness was higher in children with constipation.^10^ So we evaluated the US for the rectal measurements after the urination. In a study by Klijn et al^18^ the mean diameter of the rectum was 4.9 cm in children with constipation and 2.1 cm in a control group. In a different study, rectum diameters were measured from 3 different areas: the symphysis pubis, under the ischial spine, and at the bladder neck. It was found that the symphysis pubis, ischial spine, and bladder neck measurements of children with fecal retention were significantly higher than that of children without fecal retention. To define fecal retention, the cut-off value for the rectal diameter measured at the symphysis was taken as 27 mm with high sensitivity and specificity (95.5% and 94.1%, respectively). These authors concluded that rectal diameter measurement at the symphysis pubis by US is useful for detecting fecal retention easily and accurately.^7^ In our study, at the symphysis pubis plane, the rectal diameter measurement of constipated patients with fecal mass positive was found to be significantly greater in groups II and III than in the control group. Significantly higher values were obtained from the ischial spine planes measurements in children with fecal mass positive in group III compared to the control group. These results were similar to other studies in the literature.^7,10^

Measurement of rectal diameter based on age was evaluated for the first time in a study from Poland. The patients were grouped as under 3 years old, 3-6 years old, 6-12 years old, and over 12 years old and were compared with control group patients of the same ages. It was determined that the rectum diameter values of the constipated groups of all ages were significantly higher than those of the control groups and that the difference was most prominent in children under 3 years of age. As the patients got older, the difference between them was smaller but still significant.^12^ Doniger et al^19^ and Pop et al^20^ found a strong correlation between enlarged transrectal diameter and constipation. When rectal diameter was measured in the axial plane, it was found to be 31.72 ± 6.93 mm in the constipated patient group and 19.85 ± 4.37 mm in the control group (*P* = .001).^1^ In these studies, the patient groups were not divided into subgroups according to the presence or absence of fecal mass. Since rectal diameter values are affected by defecation and fecal retention,^9^ we evaluated study groups’ measurements made by dividing them into subgroups according to the presence or absence of stool mass in the rectum. The detailed evaluation of the data in this way makes our study different from other similar works to date. In our study, we found that the rectum diameter values measured from all planes with fecal mass positive groups were statistically significantly higher in the group aged 6.1-12 years than the control groups. We also found that the symphysis pubis and bladder neck planes measurements of children aged 6.1-12 years with fecal mass negative groups were statistically significantly higher than the control group. Fecal mass positive or negative constipated groups’ mean rectal diameter measurements increased with age, and this finding was compatible with the literature.^12^ In another study, re-evaluated rectal diameters after constipation treatment showed that measurements decreased after 4 weeks of polyethylene glycol treatment.^20^ However, we could not re-evaluate our patients’ rectal diameter measurements after constipation treatment.

Berger et al^8^ reported that they could not show a relationship between the clinical findings of constipation such as constipation duration, fecal retention, and fecal incontinence and ultrasonographic rectal diameter measurement, contrary to the literature data. In our study, we found that as the duration of constipation increased, the anterior rectum wall thickness increased. Contrary to the data of our study, in another study, the rectum wall thickness measurement of the constipated group was found to be lower. In that study, the correlation between constipation duration and anterior rectum wall thickness was not investigated.^1^ In addition, the difference in the anterior rectal wall thickness compared to the control group in that study may be due to the different constipation durations of the children in the patient group. In our study, anterior rectum wall thickness was higher in the constipated group with fecal mass positive compared to the control group only in group III, while it was statistically significantly higher in the constipated group with fecal mass negative in group II, group III, and group IV. The fact that the anterior rectal wall thickness of constipated children older than 3 years, which was measured when the rectum was empty, was statistically significantly higher than in non-constipated children suggests that it could be a useful measurement as a marker of chronic constipation.

This study is the first study in which different planes of measurements of rectal diameter and anterior wall thickness were evaluated in detail in groups with fecal mass presence or absence among constipated and healthy children in 4 different age groups. In addition, the number of patients is higher than in other studies conducted on this subject so far, and it is an important study in terms of determining the mean rectal diameter measurements and mean anterior wall thickness values of children in certain age ranges.

The limitations of our study are that we could not give a cut-off value for rectal diameter and anterior wall thickness due to the low number of children in the subgroups of the study. Due to the small number of children in the subgroups, we could not detect changes in rectal diameters related to gender. Another limitation is not being able to re-evaluate the rectum diameters of constipated patients after treatment. The lack of age- and gender-matched control group and the measurements not being measured by a single radiologist are other limitations of our study.

The measurement made under the ischial spine plane in children aged 6.1-12 years using the US technique may be useful in the diagnosis of chronic constipation. Rectal anterior wall thickness measurement using the US technique is a noninvasive method performed in children who are older than 3 years while the rectum is empty and who do not want to have a digital rectal examination.

## Figures and Tables

**Table 1.** Demographic Features Among Constipation and Control Groups

	Constipated (n = 140) Group I (n = 33) Group II (n = 23) Group III (n = 49) Group IV (n = 35)	Control (n = 164) Group I (n = 33) Group II (n = 30) Group III (n = 51) Group IV (n = 50)	*P*
Sex (M/F)(n%)	62 (44.3)/78(55.7)	68 (41.5)/96(58.5)	.62
Mean age, years	8.1 ± 5.2	8.5 ± 5.2	.53
Mean weight, kg	30.74 ± 19.33	31.81 ± 19.15	.62
Weight percentile	43.13 ± 33.39	43.52 ± 31.37	.91
Mean height, cm	123.23 ± 31.84	126.81 ± 31.56	.32
Height percentile	45.43 ± 31.69	49.13 ± 31.32	.38

**Table 2.** Comparison of the Rectal Diameter Values Measured Above the Symphysis Pubis

Groups (Ages)	Presence of Fecal Mass Rectal Diameter (Mean ± SD)		*P*	Absence of Fecal Mass Rectal Diameter (Mean ± SD)		*P*
	Constipated	Control		Constipated	Control	
Group I (≤3 years)	24.45 ± 7.89	24.26 ± 8.43	.94*	18.09 ± 4.67	16.83 ± 4.43	.47*
Group II (3.1-6 years)	26.93 ± 5.72	22.00 ± 6.93	**.04** *	18.57 ± 5.31	17.60 ± 2.79	.57*
Group III (6.1-12 years)	33.41 ± 7.21	27.55 ± 7.11	.003**	23.55 ± 8.19	19.20 ± 3.64	.05**
Group IV (>12 years)	32.00 ± 6.03	33.16 ± 6.79	.59**	22.80 ± 6.30	22.76 ± 5.22	.98**

*t-test, **Mann–Whitney *U* test.

SD, standard deviation.

**Table 3.** Comparison of the Rectal Diameter Measured Under the Ischial Spine

Groups	Presence of Fecal Mass Rectal Diameter (Mean ± SD)		*P*	Absence of Fecal Mass Rectal Diameter (Mean ± SD)		*P*
	Constipated	Control		Constipated	Control	
Group I (≤3 years)	11.22 ± 2.13	12.60 ± 3.68	.16*	9.63 ± 1.36	9.94 ± 1.73	.62*
Group II (3.1-6 years)	12.18 ± 4.05	11.00 ± 1.96	.31*	10.00 ± 3.69	9.80 ± 2.98	.89*
Group III (6.1-12 years)	14.58 ± 5.18	12.18 ± 2.74	.03**	11.94 ± 2.73	10.33 ± 1.65	.04**
Group IV (>12 years)	14.42 ± 2.47	15.75 ± 3.56	.22**	13.28 ± 4.80	12.11 ± 2.25	.31**

*t-test, **Mann–Whitney *U* test.

SD, standard deviation.

**Table 4.** Comparison of the Rectal Diameter Measured at the Bladder Neck

Groups	Presence of Fecal Mass Rectal Diameter (Mean ± SD)		*P*	Absence of Fecal Mass Rectal Diameter (Mean ± SD)		*P*
	Constipated	Control		Constipated	Control	
Group I (≤3 years)	18.00 ± 6.00	17.80 ± 4.91	.92*	13.68 ± 3.40	12.33 ± 2.22	.21*
Group II (3.1-6 years)	19.03 ± 3.96	15.86 ± 4.70	.05*	12.42 ± 2.99	12.40 ± 1.63	.98*
Group III (6.1-12 years)	23.74 ± 5.71	18.74 ± 4.94	.001**	16.11 ± 4.08	14.45 ± 3.42	.16**
Group IV (>12 years)	19.71 ± 6.92	22.25 ± 5.31	.25**	17.71 ± 6.29	17.30 ± 4.63	.80**

*t-test **Mann–Whitney *U* test.

SD, standard deviation.

**Table 5.** Comparison of the Rectal Anterior Wall Thickness

Groups	Presence of Fecal Mass		*P*	Absence of Fecal Mass		*P*
	Constipated	Control		Constipated	Control	
Group I (≤3 years)	1.52 ± 0.26	1.57 ± 0.34	.59*	1.35 ± 0.45	1.28 ± 0.17	.59*
Group II (3.1-6 years)	1.72 ± 0.56	1.45 ± 0.21	.09*	1.67 ± 0.42	1.34 ± 0.22	.02*
Group III (6.1-12 years)	2.24 ± 0.84	1.54 ± 0.34	.000**	2.52 ± 0.95	1.59 ± 0.26	.001**
Group IV (>12 years)	2.19 ± 0.70	1.85 ± 0.38	.06**	3.14 ± 1.43	1.79 ± 0.27	.000**

*t-test **Mann–Whitney *U* test.
